# A minute focus of extranodal marginal zone B-cell lymphoma arising in Hashimoto thyroiditis diagnosed with PCR after laser capture microdissection: a case report

**DOI:** 10.1186/1756-6614-2-9

**Published:** 2009-09-07

**Authors:** Antonio D'Antonio, Alessia Caleo, Stefano Licci, Maria Addesso, Maurizio De Palma, Amedeo Boscaino, Oscar Nappi

**Affiliations:** 1 Unit of Pathology, OO.RR. "San Giovanni e Ruggi d'Aragona", Salerno, Italy; 2 Department of Pathology, "Santo Spirito" Hospital, Rome, Italy; 3 Department of Pathology, "ASL SA1" Scarlato Hospital, Scafati (SA), Italy; 4 Department of Endocrine Surgery, "A. Cardarelli" Hospital, Naples, Italy; 5 Department of Pathology, "A. Cardarelli" Hospital, Naples, Italy

## Abstract

**Background:**

Primary thyroid gland lymphomas are uncommon tumours that occur in the setting of lymphocytic thyroiditis or Hashimoto's disease in almost all cases. In this condition a distinction between an inflammatory lymphoid infiltrate and a low grade lymphoma may be extremely difficult and precise criteria are necessary for a correct diagnosis.

**Patient and methods:**

We report a case of a minute focus of primary extranodal marginal zone B-cell lymphoma (EMZBCL), incidentally discovered in a 63-year-old man with Hashimoto thyroiditis (HT) and diagnosed by means of polymerase chain reaction (PCR) after laser capture microdissection.

The histological examination of surgical specimen confirmed the diagnosis of HT and showed a minute focus of dense lymphoid infiltrate (less than 4 mm in diameter), composed by centrocyte-like cells forming MALT balls. Immunoistochemistry was not useful. A microscopic focus of EMZBCL was suspected on the basis of morphological features. PCR assays revealed the rearrangement of the heavy chain of immunoglobulins only in the microdissected suspicious area, confirming the diagnosis of EMZBCL.

**Conclusion:**

Our finding suggests that in cases of autoimmune thyroiditis a careful examination of the thyroid specimen is warranted, in order to disclose areas or small foci of lymphomatous transformation. Furthermore, in difficult cases with doubtful immunohistological findings, ancillary techniques, such as molecular studies, are necessary for a conclusive diagnosis.

## Introduction

Extranodal marginal zone B-cell lymphoma (EMZBCL) mucosa-associated lymphoid tissue (MALT)-type frequently occurs in stomach, salivary glands, lung and breast. Primary involvement of thyroid gland is rare, usually arising in the setting of a lymphocytic thyroiditis [1-4].

In such cases, EMZBCL clinically appears as a gradual diffuse enlargement of the thyroid gland or as a slowly growing nodule in patients with long-standing Hashimoto thyroiditis (HT) [1]. The diagnosis of EMZBCL in the background of a diffuse inflammatory lymphoid infiltrate may be extremely difficult on routinely examined histological sections. We report the case of a minute focus of EMZBCL of the thyroid gland, diagnosed by means of polymerase chain reaction (PCR) after laser capture microdissection (LCM).

## Case presentation

A 63-year-old man presented with a clinical history of goiter and dysphagia. Physical evaluation revealed an enlarged and firm thyroid gland. No lymphadenopathy was recorded. The ultrasound scan showed a diffuse enlargement of the gland, with no calcification. The thyroid function tests showed a primary hypothyroidism with high serum titers of anti-thyroglobulin and anti-microsomal antibodies. One week after admission the patient underwent a total thyroidectomy because of increasing dyspnea and dysphagia.

Grossly, the thyroid gland was diffusely enlarged and showed a vaguely lobulated, pale, white-tan cut surface, with no macroscopic distinct nodules. Sections from paraffin-embedded tissue taken initially from 15 different levels of the gland parenchyma were examined.

**Figure 1:**
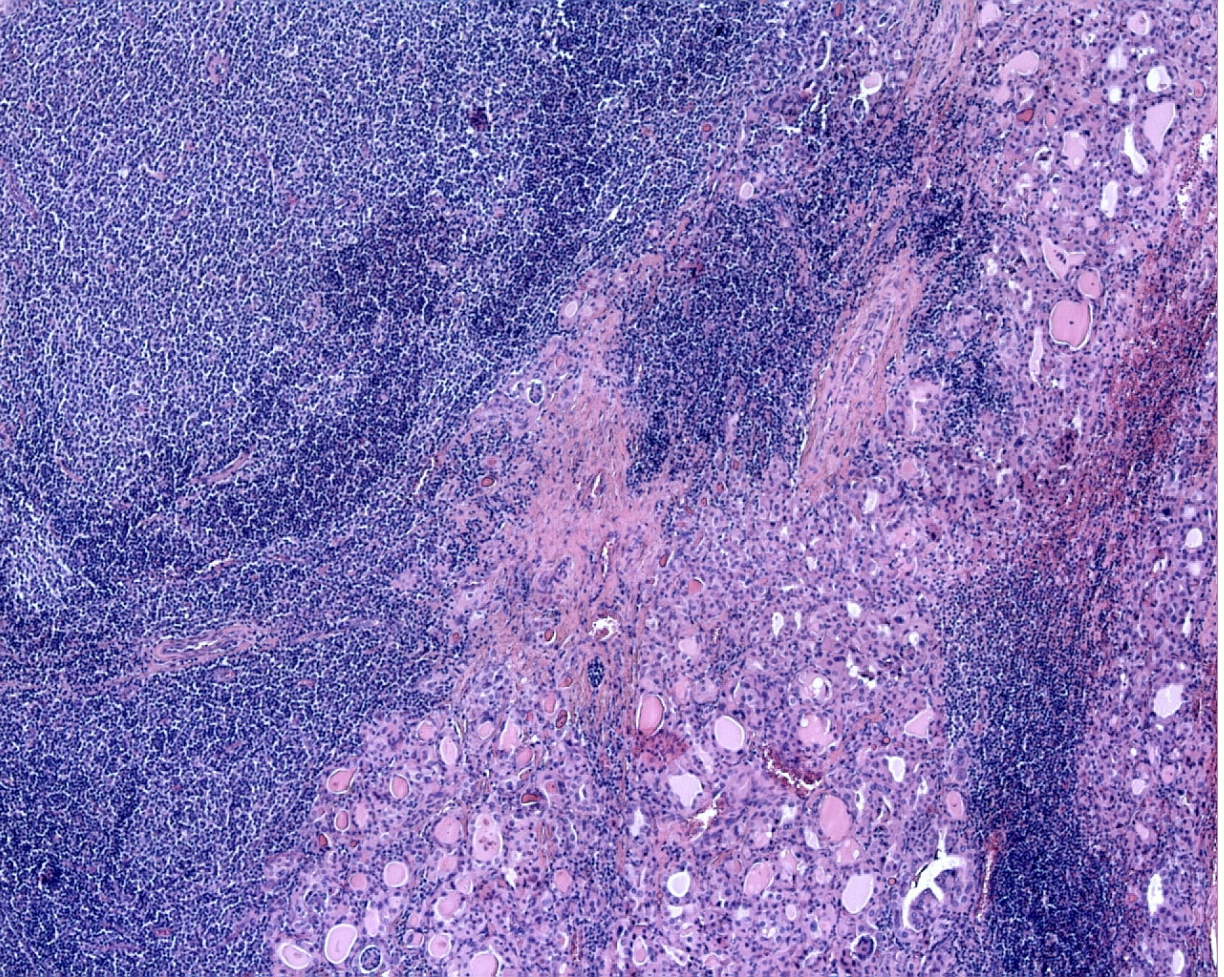
**Low power examination shows a diffuse effacement of the thyroid parenchyma by a dense lymphoid infiltrate (hematoxylin-eosin, original magnification 10×)**.

**Figure 2:**
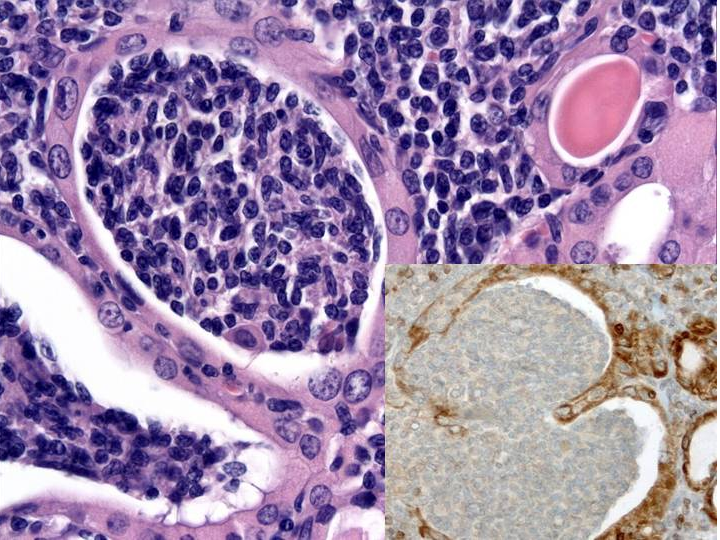
**An important diagnostic feature for the morphological diagnosis of lymphoma is the presence of lymphoepithelial lesions with packing of follicular lumens by centrocyte-like lymphoid cells (MALT-balls) (hematoxylin-eosin, original magnification 40×)**. This feature is highlighted by cytokeratins immunostain of epithelial follicular cells (inset, original magnification 40×).

The histological findings were typically indicative of HT. In the background composed by small lymphocytes, plasma cells, lymphoid follicles and oncocytic cells, we incidentally discovered a minute area, less than 4 mm in diameter, characterized by a diffuse and dense lymphoid infiltrate, composed by small lymphocytes and centrocyte-like lymphoid cells with slightly irregularly folded nuclei (Fig. 1). A few large cells were also present. Centrocyte-like cells showed a tendency to invade and expand the thyroid follicles forming MALT-balls, highlighted by cytokeratins immunostain of epithelial follicular cells (Fig. 2).

**Figure 3:**
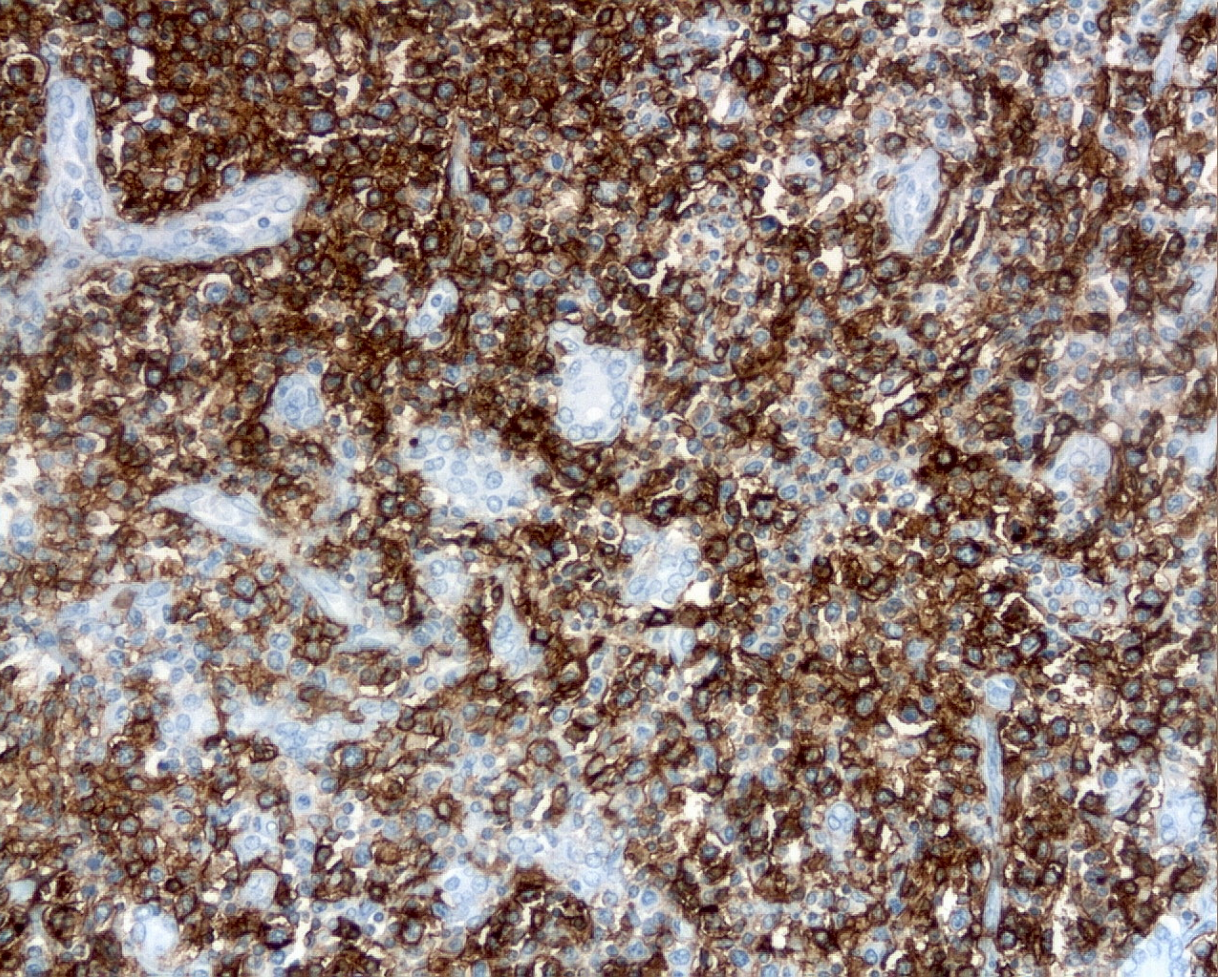
**Lymphomatous cells show a strong positivity for CD20 immunostain (original magnification 20×)**.

The immunohistochemical study showed positivity of lymphoid cells for the B cell-lineage marker CD20 (Fig. 3); CD5, CD10, bcl-6, CD23, CD43, cyclinD1 and bcl-2 were negative with expression of Ki67(MIB-1) in less than 5% of neoplastic cells. No immunoglobulin light chain restriction was demonstrated.

25 more sections were taken from the surgical specimen, including all the areas macroscopically suggestive of lymphoid tissue, and histological examination on sections obtained from paraffin blocks at different levels did not show further areas or minute foci histologically consistent with lymphomatous transformation.

A minute focus of EMZBCL was suspected only on the basis of morphology.

**Figure 4:**
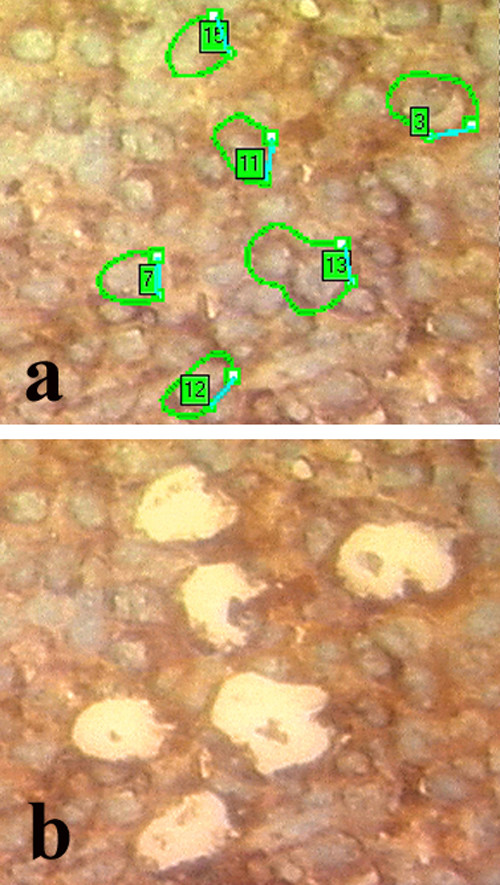
**Images of laser microdissection procedure**. A lymphomatous area is identified and selected (a) in the context of the thyroid gland section, for microdissection (b) and subsequent PCR studies (CD20 immunostain, original magnification 40×).

**Figure 5:**
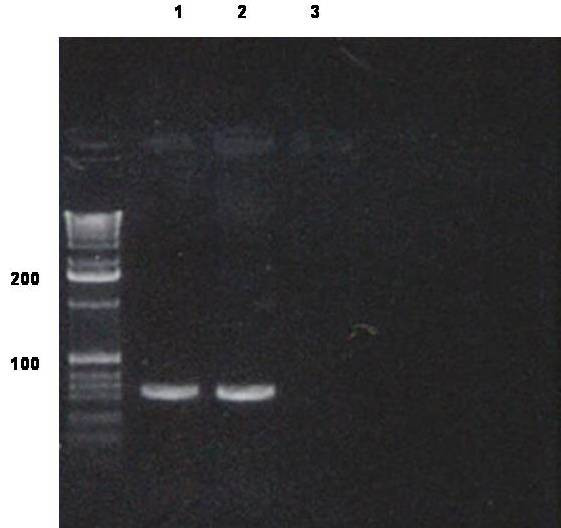
**Polyacrylamide gel electrophoresis of Ig heavy chain after PCR amplification of VDJ gene: lane 1, positive control; lane 2, our case (microdissected areas); lane 3, negative control**.

For molecular studies, we identified on hematoxylin-eosin and CD20 immunostained sections areas composed only by lymphomatous tissue and large lymphoid cells, and isolated them by LCM (Fig. 4), with subsequent DNA extraction for assessment of B cell clonality, as extensively previously described [5,6]. PCR assays, using published consensus primers, showed a immunoglobulin heavy chain rearrangement (VDJ), demonstrating the B cell monoclonal nature of lymphoid cells (Fig. 5).

Post-surgery general staging, including CT scan, did not document other pathological findings. A final diagnosis of primary EMZBCL MALT-type arising in HT was made. After surgery, the symptoms disappeared; the patient subsequently underwent only hormonal substitutive therapy and has been symptom-free for ten months.

## Discussion

EMZBCL is a low-grade lymphoma arising in MALT, originally described in the gastrointestinal tract, subsequently encountered in a variety of organs, such as lung, breast, salivary and lacrimal glands and thyroid [7-10]. In these sites, MALT appears as result of specific immune reactions or autoimmune disease [7-11].

In fact, lymphocytic thyroiditis or HT are almost certainly a requisite for development of EMZBCL in the thyroid gland [3]. EMZBCL is an indolent tumour which tends to remain localized in the thyroid and the lymphomatous infiltrate is often focal in a background of thyroiditis. In these cases a distinction between EMZBCL and diffuse inflammatory lymphoid infiltrate may be extremely difficult and strict criteria are necessary for the differential diagnosis. Histologically, the presence of a dense lymphoid infiltrate with centrocyte-like cells forming MALT-balls may be suggestive but not specific of lymphoma. The presence of plasmacytoid cells with light chain immunoglobulin restriction is a very important finding for a diagnosis of lymphoma, but represents an inconstant feature. In difficult cases the molecular study for IgH gene rearrangements, demonstrating the B cell monoclonal nature of lymphoid cells, supports a correct diagnosis of lymphoma [6].

In our case the presence of a minute focus of 4-mm in diameter incidentally discovered after an extensive sampling of the entire thyroidectomy specimen represents an effective potential diagnostic pitfall.

PCR assays revealed the rearrangement of IgH only in the microdissected lymphomatous cells confirming the morphological diagnosis of EMZBCL.

The minute focus of EMZBCL, arising in HT and diagnosed by means of molecular assays, probably represents an early-stage in the development of a low-grade lymphoma strictly correlated to autoimmune thyroiditis. This appears an additional confirmation of the hypotesis that primary thyroid EMZBCL may evolve from HT [3,12].

Thyroid EMZBCL is an indolent lymphoproliferative disorder and, when other localizations of disease can be excluded, total thyroidectomy, followed by radiotherapy when appropriate, represents a curative surgical treatment with a very good prognosis [2]. Nevertheless, the disclosure of incidental lymphomatous areas is important to assure an accurate follow-up programme for the lymphoproliferative disease, as well as for autoimmune disorders.

The purpose of this report is to underline the difficulties in the formulation of a differential diagnosis, also for an experienced pathologist, in cases of lymphoma arising in a background of HT. Both morphology and immunohistochemistry can result ineffective. Nevertheless, also in presence of a disease incidentally discovered or with an indolent behaviour, the pathologist has the obligation to furnish a correct diagnosis. Then, in difficult cases with doubtful immunohistological findings, ancillary techniques, such as molecular studies, are necessary for a conclusive diagnosis.

## Consent

Written informed consent was obtained from the patient for publication of this case report and accompanying images. A copy of the written consent is available for review by the Editor-in-Chief of this journal.

## Competing interests

The authors declare that they have no competing interests.

## Authors' contributions

AD, AC, MA, SL carried out the molecular studies and drafted the manuscript. SL carried out the immunoassays. AD, AC, MA, AB participated in the design of the study. AD, MD conceived of the study, and participated in its design and coordination. All authors read and approved the final manuscript.
